# Update on Commonly Used Molecular Typing Methods for *Clostridioides difficile*

**DOI:** 10.3390/microorganisms11071752

**Published:** 2023-07-05

**Authors:** Ana Abad-Fau, Eloísa Sevilla, Inmaculada Martín-Burriel, Bernardino Moreno, Rosa Bolea

**Affiliations:** 1 Departamento de Patología Animal, Facultad de Veterinaria, Instituto Agroalimentario de Aragon-IA2-(Universidad de Zaragoza-CITA), 50013 Zaragoza, Spain; anaabad@unizar.es (A.A.-F.); bmoreno@unizar.es (B.M.); rbolea@unizar.es (R.B.); 2 Centro de Encefalopatías y Enfermedades Transmisibles Emergentes, Facultad de Veterinaria, Universidad de Zaragoza, 50013 Zaragoza, Spain; minma@unizar.es; 3 Laboratorio de Genética Bioquímica, Facultad de Veterinaria, Instituto Agroalimentario de Aragon-IA2-(Universidad de Zaragoza-CITA), 50013 Zaragoza, Spain

**Keywords:** *C. difficile* epidemiology, MLST, MLVA, molecular typing, PFGE

## Abstract

This review aims to provide a comprehensive overview of the significant *Clostridioides difficile* molecular typing techniques currently employed in research and medical communities. The main objectives of this review are to describe the key molecular typing methods utilized in *C. difficile* studies and to highlight the epidemiological characteristics of the most prevalent strains on a global scale. Geographically distinct regions exhibit distinct strain types of *C. difficile*, with notable concordance observed among various typing methodologies. The advantages that next-generation sequencing (NGS) offers has changed epidemiology research, enabling high-resolution genomic analyses of this pathogen. NGS platforms offer an unprecedented opportunity to explore the genetic intricacies and evolutionary trajectories of *C. difficile* strains. It is relevant to acknowledge that novel routes of transmission are continually being unveiled and warrant further investigation, particularly in the context of zoonotic implications and environmental contamination.

## 1. Introduction

### 1.1. Purpose of Work

The objectives of this review are to describe some of the most important molecular typing techniques currently used in the *Clostridioides difficile* research and medical community and to highlight the epidemiological characteristics of the most prevalent strains worldwide. In addition, this review will also explore prospective research directions in the field that will allow to enhance our understanding of this intricate bacterium.

### 1.2. Clostridioides difficile Infection

*Clostridioides difficile*, formerly known as *Clostridium difficile* [1], is an anaerobic, Gram-positive bacterium known for its slow growth and unique characteristics. Most strains are capable of producing two major toxins: toxin A and toxin B. These toxins exhibit potent cytotoxic effects on host cells and are encoded by *tcdA* and *tcdB* genes, respectively, located in a locus of pathogenicity (PaLoc) [2]. Some strains are also capable of producing a binary toxin (CDT), but its role in the disease development is still uncertain [3].

This bacterium is primarily associated with a healthcare-associated infection known as *Clostridioides (Clostridium) difficile* infection (CDI). CDI can manifest as mild to severe diarrhea and, in severe cases, as pseudomembranous colitis, which can be life-threatening. It can also have a recurrent nature, causing a significant burden on healthcare facilities and patients’ quality of life [4].

Historically, CDI was predominantly considered a nosocomial infection, acquired especially in hospitals and long-term care facilities. However, there has been a noticeable shift towards community-acquired CDI cases in the last decade [5]. Additionally, certain strains of *C. difficile* have exhibited increased virulence, leading to severe and recurrent infections. This phenomenon, often referred to as hypervirulence, has raised concerns in healthcare settings, making clear the necessity of developing more effective preventive measures [6]. Furthermore, *C. difficile* is known to be associated with antimicrobial resistance to a wide range of antimicrobial agents, further complicating treatment options and contributing to the challenges in managing CDI [7].

### 1.3. Epidemiology of CDI

*C. difficile* is recognized as the leading cause of healthcare-associated infective diarrhea, particularly among patients receiving antibiotic therapy. *C. difficile* can persist in the environment for extended periods thanks to its ability to sporulate, making contaminated surfaces and equipment potential sources of infection. It is transmitted through the fecal–oral route, with contaminated hands and surfaces serving as vehicles for transmission [8].

Several studies have identified *C. difficile* in a great variety of animal species, including livestock, pets, and wildlife [9,10,11,12,13,14]. This fact has raised concerns about potential zoonotic transmission, where animals may serve as reservoirs and a source of infection for humans through direct contact or consumption of contaminated animal products. It is believed that animals may acquire *C. difficile* through exposure to contaminated environments, contaminated food, or through contact with infected humans. Several studies have demonstrated high genetic similarities between *C. difficile* isolates from humans and animals [15,16,17], providing evidence for potential transmission between species. However, the exact mechanisms and extent of zoonotic transmission are still not fully understood and require further investigation as there are also cases that show limited risk of transmission [18,19,20]. Nevertheless, a One Health approach is needed to better understand *C. difficile* epidemiology.

## 2. Molecular Typing Techniques in *C. difficile*

In the field of *C. difficile* research and healthcare, a wide range of techniques and approaches are employed to study various aspects of this bacterium, including its virulence factors, epidemiology, and interactions with the host.

In Europe, PCR ribotyping is regarded as the preferred typing method for *C. difficile*, providing valuable information about the genetic diversity and relatedness of *C. difficile* strains and enabling effective surveillance and outbreak investigations [21]. On the other hand, in North America, pulsed-field gel electrophoresis (PFGE) is more commonly employed for molecular characterization of *C. difficile*. This technique allows the comparison of banding patterns to identify genetic variations among strains [22].

**Figure 1:**
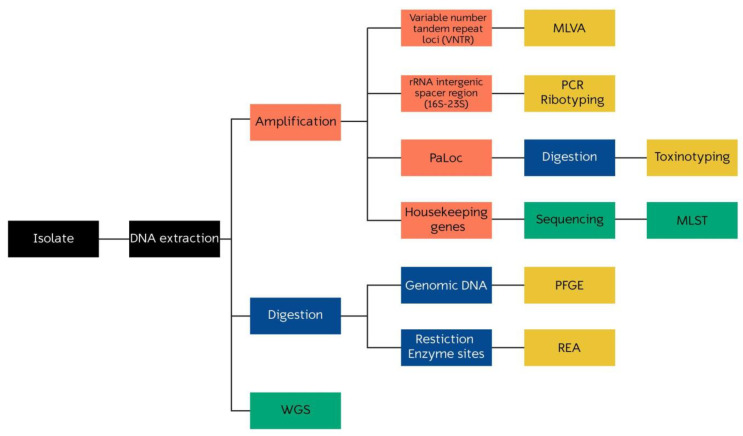
Overview of the protocols of molecular typing techniques in *C. difficile*. Each color represents a different technique: blue for enzymatic digestion, orange for PCR amplification, green for DNA sequencing, and yellow for electrophoresis and pattern analysis.

While PCR ribotyping and PFGE are the predominant methods in Europe and North America, respectively, it is important to note that there are other available molecular characterization techniques that provide valuable insights into *C. difficile*. Each method offers unique advantages in terms of resolution, accuracy, and the ability to detect specific genetic markers. In the following sections, we will explore a selection of the most common techniques that have greatly contributed to the understanding of *C. difficile*, considering their respective attributes and potential applications. A brief summary of each technique can be found in Table 1. In addition, Figure 1 represents a concise overview of the protocols of these techniques.

### 2.1. PCR Ribotyping

One of the widely used molecular typing techniques for *C. difficile* is PCR ribotyping. PCR ribotyping involves the amplification of a specific region of the bacterial genome, known as the 16S-23S rDNA intergenic spacer region. Each pattern of DNA fragments corresponds to a ribotype (RT), and it is based on the differences in size and distribution of the amplified fragments. This allows the differentiation and classification of *C. difficile* strains into distinct ribotypes [23]. Harmonization and standardization of this methodology have been validated by a multicenter study comprising Europe, North America, and Canada, allowing its spread as a high quality technique with comparable results [24]. Also, the existence of public databases has improved inter-center comparisons [25].

The use of capillary gel electrophoresis in PCR ribotyping, instead of the conventional agarose gel electrophoresis, has improved this technique as it offers several advantages, including a high discriminatory power, reproducibility, and ease of interpretation [23]. Ribotyping data can be easily shared among laboratories and integrated into global databases, helping the comparison and tracking of *C. difficile* strains across different regions and time periods.

Moreover, PCR ribotyping has revealed the existence of specific ribotypes that are associated with different clinical outcomes, virulence characteristics, and antibiotic-resistance profiles. Global geographical prevalence has also been previously discussed [26]. Understanding the distribution and prevalence of specific PCR ribotypes can provide valuable insights into disease transmission, the emergence of hypervirulent strains, and the impact on public and animal health.

### 2.2. Pulsed-Field Gel Electrophoresis (PFGE)

PFGE is a molecular typing method that consists of digesting bacterial DNA with a restriction enzyme, followed by separation of the resulting fragments by electrophoresis using an alternating and cross field, which allows the separation of large DNA fragments [27,28]. PFGE was adopted as the primary typing method for *C. difficile* by both CDCs from Canada and the USA, although it was later on substituted by other methods such as MLST or WGS [29,30].

PCR ribotyping and PFGE have been the most popular methods for strain characterization. Despite having similar utilities and a good correlation [31], PFGE can discriminate some strains better than ribotyping [32], while it is complicated to be used in others such as RT 1 strains [33], although this issue has been already investigated and solved through an increase of lysis time, lysozyme concentration, and proteinase K, among other changes in protocol [34].

PFGE has been used to study the epidemiology and transmission of *C. difficile* as it can provide high-resolution molecular typing with good discriminatory power. PFGE has been shown to be effective in identifying outbreaks and tracking transmission of *C. difficile* strains within hospitals and other healthcare facilities [35].

However, PFGE has some limitations, including the requirement for highly skilled personnel and specialized equipment as well as potential technical variability between laboratories. Additionally, PFGE results can be difficult to interpret due to the high degree of genetic diversity within *C. difficile* strains, which can make it challenging to establish standardized interpretation criteria.

Overall, although PFGE has been a valuable tool for studying the epidemiology of *C. difficile*, like other techniques its use is being reduced in favor of newer, higher-resolution molecular typing methods such as whole-genome sequencing [28].

### 2.3. Restriction Endonuclease Activity (REA)

Restriction endonucleases are enzymes that cleave DNA molecules at specific recognition sites, resulting in fragments of different lengths. The REA technique involves the digestion of DNA with one or more restriction enzymes, followed by separation of the resulting fragments using gel electrophoresis. By comparing the DNA fragment patterns of different samples, REA can be used for a variety of applications, such as strain typing, identification, and phylogenetic analysis [36]. An extensive protocol was developed by Clabots et al. [37] and showed reproducible results consistent with other typing methods. However, the highly demanding protocol made it a technique rarely used in other laboratories.

Also, REA has been used as a way to determine relatedness in the case of outbreaks as it is capable of distinguish between strains of the same type, as opposed to ribotyping or PGFE [37]. REA and PFGE have been shown to have similar discriminatory power and higher than PCR ribotyping [38].

Although REA analysis is a useful technique for studying the epidemiology of *C. difficile* and management of hospital outbreaks, it has not been widely used within the *C. difficile* research community [39].

There is little information on REA profiles, especially when compared with other techniques. In general, most studied RT 027/NAP1 strains are associated with REA group BI, while other important RT 078-like strains are linked to REA group BK, and RT 014/020 is linked to group Y [40]. The strong correlation between ribotyping and REA suggests that the results obtained through ribotyping may provide valuable insights into REA analysis [41].

It should be noted that REA requires a high manual labor as well as involves subjectivity in interpretation of the banding patterns in the gel. This along with the lack of standardization and its cost may be the reasons why this kind of analysis lacks popularity within the *C. difficile* research community.

### 2.4. Multilocus Variable-Number Tandem-Repeat Analysis (MLVA)

In MLVA, a set of predetermined genetic loci are selected for analysis. These loci consist of repeated DNA sequences where the number of repeats varies between strains of *C. difficile*. The variations in the number of repetitions are measured using PCR amplification and fragment analysis techniques [42].

By comparing the lengths of the amplified fragments in the loci among different *C. difficile* strains, MLVA generates unique genetic profiles for each strain, which can be used to differentiate and classify different isolates, allowing for strain identification, tracking of outbreaks, and understanding the genetic relatedness among isolates.

MLVA has emerged as a valuable technique for differentiating strains during CDI outbreaks. It has been described that MLVA, as well as REA, offer high resolution in genotyping, while other methods fail.

To ensure the comparability of MLVA results across different laboratories, the establishment of standardized protocols and the use of reference strains as controls are essential. This will promote consistency and reproducibility of MLVA analysis across laboratories, ensuring the reliability of the technique.

In summary, MLVA has demonstrated its effectiveness in characterizing and subtyping *C. difficile* strains during outbreaks. Its ability to provide high-resolution genotyping along with its simplicity and cost-effectiveness make it a valuable tool in the study of *C. difficile* epidemiology, especially since it is easily standardizable and results can be shared between laboratories. As research progresses and technologies advance, the integration of MLVA with other molecular techniques, such as genome sequencing, may further enhance our understanding of *C. difficile* strains and their transmission dynamics.

### 2.5. Toxinotyping

Another historically relevant typing technique, although less used nowadays, is toxinotyping. This method is based on the use of PCR-restriction fragment length polymorphisms to assess the differences in the PaLoc region [43].

The significance of toxinotyping lies in its ability to characterize the genetic variability of *C. difficile* toxins, particularly toxins A and B, which are considered the primary virulence factors and play crucial roles in the pathogenesis of *C. difficile*-associated diseases. Toxinotypes, therefore, represent a diverse group of strains that exhibit alterations in the toxin A and B coding regions. The differences in the patterns after the use of restriction enzymes, and its comparison with standardized patterns, allow the assignment of the strains into one of the 34 toxinotypes, which are labeled from I to XXXIV in Roman numerals.

The toxinotyping method was initially described in 1998 and has since undergone updates to incorporate new knowledge and advances in molecular techniques [44]. It provides a reliable and standardized approach for characterizing C. difficile strains based on toxin gene variations, facilitating epidemiological investigations, and informing the development of preventive strategies. The continuous discovery of new toxinotypes and improvements in toxinotyping protocols contribute to our comprehensive understanding of the genetic landscape and pathogenic potential of *C. difficile*.

### 2.6. Multilocus Sequence Typing (MLST)

MLST is a molecular typing method that studies molecular diversity among different strains of a bacterial species. It is based on the sequence of a selected set of housekeeping genes, which in the case of *C. difficile* are the following: *adk* (adenylate kinase), *atp*A (ATP synthase subunit alpha), *dxr* (1-deoxy-d-xylulose 5-phosphate reductoisomerase), *gly*A (serine hydroxymethyl transferase), *rec*A (recombinase A), *sodA* (superoxide dismutase), and *tpi* (triose phosphate isomerase) [45]. These genes are present in all strains of *C. difficile* and can be therefore compared between strains. When these gene sequences are compared, allelic profiles are generated based on the specific variants found at each gene. These allelic profiles are then used to define sequence types (STs), which identifies each strain. A curated database can be found at https://pubmlst.org/organisms/clostridioides-difficile (accessed on 30 May 2023), which allows comparisons between laboratories [25,45].

This tool has been used to track epidemiology changes in bacterial populations, allowing the identification of clonal complexes and the tracing of their spread over time and across geographical locations. MLST data can be used not only to identify the strains but also to infer the evolutionary relationship among strains and to investigate the mechanisms of bacterial adaptation and evolution.

Studies have shown that the majority of STs in *C. difficile* are in accordance with their RTs [46]. Therefore, MLST and ribotyping can provide complementary information that can be used to identify and track the spread of *C. difficile*. Exploring the prevalence and distribution of *C. difficile* ribotypes has been a topic of recent interest, with a particular focus on the most common ribotypes as well as emerging ones, and the revisions on the topic can be found elsewhere [47]. The relationship between ST and RT of some of the most important *C. difficile* strains can be found in Table 2.

Based on MLST, the population structure of *C. difficile* can be divided into five distinct clades and three cryptic ones, each of them with its own characteristics [62]. Clade 1 represents the largest and most heterogeneous group, displaying significant genetic diversity within its members. Clades 2 and 5, which have been identified as harboring hypervirulent strains, have lower recombination rates compared to the other clades. In general, the recombination rates of *C. difficile* are moderate [65]. Clade 3 has garnered attention due to its unique characteristic of harboring a clade-specific PaLoc with a *Tn6218* insertion, which serves as a distinguishing feature from other *C. difficile* strains [54]. Additionally, Clades 3 and 5 share genetic similarities in their *tcdC* sequences [48].

Clade 4 is distinguished from the rest by its high prevalence of multidrug resistance (MDR) [66]. Clade 5 encompasses various strains, including those associated with animals, such as ST 11 strains. This clade has been previously associated with community-acquired *C. difficile* infection (CA-CDI). Notably, when considering long-term outcomes, Clades 2 and 5 consistently exhibit higher mortality rates, while Clades 1 and 3 display lower mortality rates, even after adjusting for differences in biomarkers [67].

More recently, cryptic Clades C-I, C-II, and C-II have been described and are associated with atypical variants of all four toxin-related genes (*tcdA*, *tcdC*, *cdtA*, *cdtB*), although they have been poorly investigated thus far [68].

It has been reported that MLST also fails to identify the problematic RT 014/020 [69] and to correctly assign phylogenetic relationship between some Clade 1 and 2 isolates [70]. Clade 4 has also presented problems in China [71] as well as some discordances in ST 3 [46,72]. The interpretation of MLST profiles can potentially lead to the erroneous diagnosis of reinfection as the identification of the same ST within the same patient does not invariably indicate relapse [73]. On the contrary, in RT 015 strains, MLST can further identify the differences between ST 44 and ST 10, and a lower mortality has been described in ST 44 [67]. Among the limitations of MLST, some clades have more differences between them than others. Clades 3 and 4 have shown to have a high inter-RT allele differences, while Clades 1 and 5 have lower [74].

Hence, it should be noted that the homology between STs and RTs is not always perfect. Some RT correlate with more than one ST, and vice versa. In this regard, MLST has been proposed as a tool for strains identification in cases where ribotyping fails to fully detect differences.

Based on this sequencing methodology, core genome (cg-) MLST and whole genome (wg-) have been also developed, using NGS technology. CgMLST is a variant of the traditional MLST technique that focuses on the sequencing of the core genes within bacterial genomes. The core genome refers to the set of genes that are present in all members of a given bacterial species and are typically conserved in their sequence and function. In opposition to the traditional approach, more information is generated and analyzed. Compared to traditional MLST, cgMLST provides a higher level of discrimination between bacterial strains and can be used to identify even closely related isolates, which makes cgMLST an optimal tool for outbreak investigations. Additionally, the use of a standard cgMLST scheme across laboratories allows comparison of results and data sharing between different institutions and countries [75,76]. As with traditional MLST, limitations include, in some specific strains, not being able to distinguish between outbreak strains and regular ones, especially in ribotypes with low intra-RT allele differences. Therefore, caution should be exercised when making epidemiological affirmations based on that information alone [74].

A few hash-based methods have been proposed based on cg MLST [25,77,78,79,80]. This type of method involves generating unique numerical identifiers, or “hashes,” for each allele in the targeted genes, instead of focusing on the core genome. These hashes are generated by converting the nucleotide sequence of each allele into a numerical value using a hashing algorithm.

In opposition to cgMLST, wgMLST analyses both the core genome and accessory genes providing a comprehensive view of its genetic building. It has the ability to detect variations that can be missed by cgMLST, giving a more accurate vision of genetic relatedness between strains. However, it should be noted that the computational power needed is much higher, and standardization between studies is harder. By tracking the genetic changes that occur over time, wgMLST can help to identify the sources of infection and the routes of transmission and can provide valuable information for infection control and prevention efforts [81,82].

When compared to other approaches, such as cgMLST and wgMLST, most strains have the same MLST profile as wgMLST. However, in certain cases, wgMLST can provide additional useful information for further strain identification and outbreak investigations, making it a valuable strategy in these scenarios [83]. As wgMLST also includes accessory genes, it can provide a higher resolution than cgMLST, being a more powerful tool when studying outbreak situations. Nevertheless, the amount of computational work needed is also much more demanding and usually does not give more detailed information than cgMLST [76,82].

### 2.7. Whole-Genome Sequencing (WGS)

WGS has been proposed as an alternative for traditional methodologies thanks to the development of next-generation sequencing techniques (NGS).

The single nucleotide polymorphism (SNP) technique has emerged as a powerful tool for molecular epidemiology and strain characterization. SNPs are single base-pair variations in the DNA sequence that can serve as genetic markers to differentiate strains and track their transmission dynamics [74].The application of SNP analysis in *C. difficile* involves sequencing and comparing specific genomic regions among different isolates. By comparing the sequence variations at specific SNP sites across the genome, the relatedness and evolutionary relationships between *C. difficile* strains can be determined.

The main advantages of SNP are its high resolution and discriminatory power, which allows the differentiation of closely related strains. It can also be used to assess the phylogenetic relationships and population structure of *C. difficile* isolates and detect genomic changes associated with virulence and antibiotic resistance [84].

However, SNP analysis requires advanced bioinformatics tools and expertise for data analysis and interpretation. Additionally, the cost and time required for sequencing whole genomes may limit its widespread adoption in routine surveillance, in addition to its difficulty in standardization. In contrast, the tools used for cgMLST typing are often user-friendly, and there is no need of selection of phylogenetically related strains to perform the analysis, which is needed for SNP typing.

As discussed previously, the use of cgMLST in investigating local *C. difficile* epidemiology has shown comparable results to SNP analysis, providing a significant advancement. The ability of cgMLST for *C. difficile* to identify closely related isolates and infer genomic distances is inferior to SNP analysis due to errors introduced during de novo assembly and a lack of per-base quality control. However, when applied to a large dataset of *C. difficile* genomes from hospital patients, both cgMLST and SNP analysis have shown to discriminate between epidemiologically related and unrelated isolates [25].

Similarly to SNP analysis, molecular sequencing studies of virulence factors have been proposed as a method for studying short-term evolution, as opposed to housekeeping MSLT techniques which are more suited to long-term evolution [85].

WGS provides a more extensive dataset compared to other types of analyses. Average amino acid identity (AAI) analysis can be performed, which compares conserved protein-coding genes among genomes. This approach clusters strains based on sharing more than amino acid content, demonstrating higher resolution at the species level compared to 16S rDNA or MLST, as it assesses a larger fraction of the genome [70]. STs and RTs do not seem to predict toxins, although toxin diversity can be predicted within the clades [86]. For this reason, toxin genetics are best investigated with WGS than with conventional approaches when gathering epidemiological information about toxins and toxin production.

Additionally, WGS has emerged as a valuable tool in approaching treatment strategies for CDI, particularly in relation to the identification of antimicrobial resistance genes. While not all resistance genes are currently known, WGS allows a comprehensive analysis of the genome, enabling the detection of known resistance mechanisms. However, genomic studies combined with phenotypic assays are still necessary to detect resistance mechanisms not described yet.

In conclusion, WGS offers valuable insights into *C. difficile* epidemiology, particularly in understanding resistance mechanisms and strain relatedness. While challenges and limitations exist, ongoing advancements in genomic analysis methods will continue to enhance our understanding of this pathogen and inform future research paths.

## 3. Molecular Epidemiology of *C. difficile*

Hospitals play a crucial role in the transmission of *C. difficile*, and extensive research has focused on unraveling the genetic profiles and transmission dynamics within healthcare facilities. Beside the nosocomial environment, CA-CDI is becoming an emerging threat. As CA-CDI refers to cases whose source of infection is not linked to healthcare facilities, they have not been as profoundly investigated as healthcare-associated CDI. Exploring the molecular epidemiology of CA-CDI helps us understand the sources and transmission routes of *C. difficile* in community settings.

Additionally, molecular epidemiology of *C. difficile* in the environment is also an interesting topic of research. This includes investigating the presence and persistence of *C. difficile* in water sources, wastewater treatment plants, and other environmental reservoirs. Understanding the genetic characteristics and behavior of *C. difficile* in the environment allows us to assess potential dissemination pathways and evaluate the associated public health risks. Furthermore, the presence of *C. difficile* in animals adds another dimension to its molecular epidemiology. The evaluation of its zoonotic potential and understanding the transmission dynamics through a One Health perspective becomes crucial.

The investigation of various isolates belonging to the same ST has revealed that antibiotic resistance and virulence factors can coexist within the same molecular profile (either ST or RT). Consequently, it is imperative to exercise caution as relying solely on molecular evolutionary distances may not be sufficient in comprehending the epidemiology of *C. difficile*. It is essential to consider other factors that may impact the pathogenesis and transmission of the bacterium. This underscores the importance of adopting a comprehensive approach that accounts for all relevant variables when studying the epidemiology of *C. difficile* [87].

### 3.1. Epidemiological Characteristics in Healthcare Setting of Commonly Isolated Sequence Types

The molecular characteristics of C. difficile strains exhibit a wide range of diversity across different countries, continents, hosts, and risk factors. A comprehensive literature review [88] provides updated insights into the geographical distribution of these strains, along with their associated characteristics, particularly focusing on commonly identified ribotypes [47]. Nevertheless, it is important to note that alternative typing methods have also yielded valuable information that may be overlooked due to their relatively lower popularity in the research community.

In studies performed in human populations, CDI cases belonging to Clade 2 were more common among older patients and those with multiple risk factors. Clade 2 and 5 infections had a significantly higher mortality rate compared to Clade 1, with a trend towards higher mortality for Clade 5 compared to Clade 2 CDI. Despite Clades 3 and 5 showing genetic similarity in several PaLoc genes, they have been found to have different mortality rates. In Clade 1, ST 44 showed a lower 14-day mortality risk, despite being highly similar to ST 10 (one nucleotide difference in the housekeeping analyzed). Both ST 10 and ST 44 are usually identified as RT 15. However, Clade 3 appeared different from other clades as it had significantly higher neutrophil, similar to Clades 2 and 5, despite significantly lower mortality [67].

One of the most common studied strains, mostly because of its hypervirulent characteristics, is known as North America PFGE type 1, or NAP-1, and is associated with certain attributes. It also correlates with RT 027 and belongs to Clade 2. NAP-1 produces high levels of toxins A and B as well as an additional binary toxin, which is linked to increased morbidity and a poor response to antibiotic treatment [89]. For these reasons, this ribotype, along with RT078, has been considered hypervirulent [90]. Furthermore, a mutation in the *tcdC* gene, a negative regulator of toxin production known as the anti-sigma factor TcdC, is also considered a key factor in the hypervirulence development of RT027 strains. This naturally occurring mutation is thought to contribute to a substantial increase in toxin production [91]. However, studies have shown that not all NAP1/027 isolates are consistently associated with a more severe disease, regardless of their ability to produce larger amounts of toxins. This suggests that other factors may also contribute to the clinical outcomes [92]. When taking into account antibiotic resistance, *C. difficile* isolates of PFGE types P1, P3, P4, P8, and P10 have been associated with high-level resistance against clindamycin, ceftriaxone, erythromycin, and ciprofloxacin [31].

In a 10-year study in a Chicago tertiary care hospital [93], they found REA technique to be able to detect clusters associated with higher incidence of *C. difficile* infection. However, despite the ever-changing strain population, some epidemic groups repeated themselves in time, concluding that the change in the bacterial population was caused by the introduction of new patients to the institution. This idea has been previously proposed in other studies including REA analysis, although there has been in general a switch to more recent techniques, such as PFGE [94].

In another study based on North America, no dominant sequence types (STs) were identified, suggesting a diverse population of *C. difficile* circulating in the healthcare facilities of both regions. The absence of large clusters of closely related isolates further suggests the effectiveness of infection control practices in preventing widespread transmission within these settings. However, different sequence types (STs) showed associations with distinct forms of CDI, with ST1, ST53, and ST43 being more likely associated with HCA-CDI and ST3 and ST41 commonly isolated from CA-CDI cases [95]. Notably, the distribution of specific STs varied between the two surveillance regions. In the northwest, ST1 was found to be more prevalent, while in Minnesota, ST41 was more commonly observed. These regional differences in ST distribution may reflect variations in the local epidemiology, patient populations, or healthcare practices. In fact, less than half of the isolates showed clear epidemiological links, indicating that a significant proportion of CDI cases may have originated from sources not directly linked to previously identified cases. This is a trend that has already been observed in other occasions [96]. The identification of diverse STs among epidemiologically unlinked isolates suggests the potential for multiple introduction events from various sources, including asymptomatic carriers and contaminated environmental reservoirs.

The distribution of *C. difficile* STs varies across continents, with some STs being more commonly isolated in certain regions compared to others. While STs 1, 2, and 3 are frequently reported across all continents, they are rarely the most commonly isolated ST in any given region [47].

For instance, in Asia, STs 37 and 54 are more commonly isolated compared to other STs. Meanwhile, in Europe, ST 11 is frequently isolated, but its presence in Asia is rarely reported [47,65,97,98,99,100]. In America, both ST 11 and ST 42 are commonly isolated [101]. ST 42 has been highly prevalent in the United States adult population. It is widely characterized by the deletion of a single nucleotide in the *tcdC* negative regulator and the lack of binary toxin. It has been associated with higher virulence. It also possesses several genes that encode proteins described in other bacterial species related to intestinal mucosal adhesion, sporulation, and protection from oxidative stress and foreign DNA [102]. ST 54 has also been found in healthy patients and in those with CDI [103]. Some STs, such as ST 37, have contradictory information. While one study showed a higher prevalence of ST 37 in diarrhea in adults but not in healthy individuals [103], in other cases, it has been found to be one of the most prevalent types in patients colonized with *C. difficile* but not presenting active CDI [104].

In a separate study, *C. difficile* ST 17 (RT 018) isolates exhibited distinct pulsotypes across different hospitals. However, when studying other hospitals, diverse STs were detected, while some types were unique to each hospital. This epidemiological study suggests that *C. difficile* infections in hospitals are associated with the persistence of endemic clones along with the emergence of unique clones. Combining MLST with PFGE or ribotyping could be a valuable approach for monitoring epidemic *C. difficile* strains and the emergence of new clones in hospital settings [105].

In addition, the application of MLVA in investigating nosocomial *C. difficile* infections has yielded promising results [106]. When analyzing isolates from several outbreaks caused by the hypervirulent RT 027 (ST 1), MLVA identified 13 distinct clusters. Additionally, MLVA analysis of 29 toxin A-negative, toxin B-positive isolates belonging to RT 017 (ST 37) from eight different countries revealed the presence of eight country-specific clusters. This highlights the ability of MLVA to subtype and differentiate newly emerging variants of *C. difficile* [42].

In regard to the relationship between non-toxigenic isolates and disease, it has been proposed that the presence of non-toxigenic *C. difficile* isolates could have a significant role in the context of CDI prevention and control [107]. These non-toxigenic strains, which do not produce the toxins associated with *C. difficile*, have emerged as potential protective agents against toxigenic *C. difficile* strains, yielding intriguing possibilities for mitigating the burden of CDI in healthcare facilities, and are seen as a possible route of study for preventive treatments in the future [108].

In conclusion, *C. difficile* strains exhibit significant diversity, and the use of various typing methods, including ribotyping, MLST, PFGE, and MLVA, has provided valuable insights into strain distribution, pathogenicity, and risk factor associations. Furthermore, the identification of specific ribotypes, such as NAP-1/027/ST2, associated with hypervirulence highlights the need for a comprehensive understanding of the complex factors contributing to disease severity. Geographic differences in strains prevalence reinforces the need of surveillance as changes in bacterial populations, specially the emergence of hypervirulent strains, can have an impact on human health. The use of alternative techniques, especially in the case of outbreaks or geographically limited locations, can provide meaningful information about transmission routes and aid in identifying potential sources of infection, such as persistent endemic clones or the emergence of unique ones.

### 3.2. Epidemiological Characteristics in Community-Acquired C. difficile Infection (CA-CDI)

In general, CA-CDI has been associated with younger patients with no prior antibiotics exposure or healthcare association [109]. This kind of patients also have a lower mortality rate compared to traditional CDI. The sources of infection can be diverse, including other humans, animals, the environment, or food sources, among others [110].

ST 11 has been associated with CA-CDI and animals. This ST has been found to contain strains from RT 078, 078-like, and 126 and also RT 045 and 033. Interestingly, Krutova et al. found that, among ST 11 isolates, only those characterized as RT 033 had a different toxigenic profile [111]. In other studies, ST 11 was found to be associated with reduced sensibility to erythromycin and moxifloxacin, but all the isolates found were from RT 78 [112].

As an example of the application of WGS in *C. difficile* epidemiology, Xu et al. [113] used geotemporal analysis to investigate strains from China. Their findings revealed that the initial introduction of ST 37 into China occurred through multiple independent importation events, and some of them likely transmitted through household acquisition. The multiple importation events from various geographic regions and subsequent household transmission and interprovincial spread suggest that CA-CDI cases in China may involve diverse sources and have the potential for widespread dissemination.

In Australia, a lower occurrence of healthcare-associated *C. difficile* infection (HA-CDI) was observed compared to surveillance reports from North America and Europe. Seasonal patterns were apparent, with declining rates of HA-CDI observed during the analyzed period. Furthermore, severe infections were more prevalent in community-associated CDI (CA-CDI), underscoring the need for heightened surveillance in community settings in the future as the cause of this increase in severity when compared to other locations is still not understood [114]. Similar findings have been found in other countries in Europe [115,116]. In America, hypervirulent NAP1 (ST 2) was found more commonly in healthcare-associated cases, while in CA-CDI cases, NAP6 (ST 8) was found in almost double the cases of nosocomial CDI. Interestingly, NAP7 (ST 11/RT 078) was not significantly different in neither of the settings, although prevalence was a bit higher in the CA-CDI group [117].

ST 3 strains have been found present in abundance in healthy children but not in adults. This type of strains has been classified as non-toxigenic several times by their lack of toxigenic genes, but they are also capable of being toxigenic [103,118]. However, they have been described in cases of CA-CDI, usually associated with fluoroquinolone resistance [119].

Other ribotypes found to be associated with CA-CDI include RT 001 (ST 3), RT 002 (ST 8), RT 015 (ST 2), RT 017 (ST 37), and RT 018 (ST 17). In some cases, the introduction of a specific strain in the hospital was linked to the admission of patients with asymptomatic *C. difficile,* and, as with CA-CDI, these patients tend to be younger. RT diversity was also higher in CA-CDI, compared with HA-CDI. These findings imply variations in pathogenicity or survival fitness among distinct ribotypes as the higher proportion of prevalent ribotypes in CDI cases, specifically CA-CDI cases, implies that these specific ribotypes have characteristics that promote their ability to cause symptomatic infections [120].

In conclusion, the emergence of CA-CDI poses a significant challenge in healthcare settings and the community. The increasing incidence of CA-CDI highlight the need for heightened surveillance and preventive measures. The unique epidemiological characteristics of CA-CDI, such as patient characteristics, strain diversity, and potential for transmission in community settings underscore the importance of understanding its risk factors and transmission dynamics.

### 3.3. Epidemiological Characteristics in the Environment

The epidemiology of *C. difficile* extends beyond the clinical setting and includes its presence and dynamics in the environment. Understanding the environmental epidemiology of *C. difficile* is crucial for comprehending the transmission pathways, reservoirs, and potential sources of infection.

In a study about wastewater, ST 2 had four different RTs: 878, 879, 020, and 014. However, when analyzed by WGS, RT 878 and 879 had 0–2 SNP differences between them, suggesting a genetic relationship between both ribotypes. In the case of the other RTs, notable differences between the strains were found, despite sharing RT and ST, highlighting the differences that these methodologies can have [121].

Through whole genome comparisons, more diverse STs were found in wastewater than in the clinical setting. However, 13 STs were common to both. Additionally, highly similar isolates were identified in both clinical and wastewater samples, indicating potential extensive release of toxigenic *C. difficile* into surface waters [122].

Regarding surface water samples, another study revealed the presence of diverse RTs and STs of *C. difficile*, including both toxigenic and nontoxigenic strains. Remarkably, one isolate exhibited resistance to metronidazole, which was attributed to the presence of a plasmid called pCD-METRO [123]. Although the resistant isolate was a nontoxigenic ribotype and could not be associated with CDI, the potential transmissibility of the plasmid and metronidazole resistance phenotype could happen [124].

Furthermore, a study conducted in Iran identified toxigenic *C. difficile* (toxinotype V) in wastewater. The isolated strain showed genetic similarities to the hypervirulent *C. difficile* RT078. The presence of multidrug-resistant strains and a *C. difficile* isolate carrying a Tn*916*-like transposon in the treatment plant outlet raises concerns about the efficacy of wastewater treatment processes. Discharge of the treated wastewater into the environment could contribute to the dissemination of *C. difficile* beyond hospital settings, potentially leading to CA-CDI in human and animal populations [125]. It has also been described that *C. difficile* can survive treatment process, in addition to those isolates being associated with those found in hospitals in the same area [126].

Overall, these findings suggest that *C. difficile* can survive the treatment processes of wastewater and be released into the environment, serving as a potential source and reservoir for CA-CDI. The overlap between *C. difficile* genotypes found in wastewater and those isolated from hospital patients indicates a possible connection and highlights the importance of understanding the environmental epidemiology of *C. difficile* for effective prevention and control strategies.

### 3.4. Epidemiological Characteristics in Animals

As a bacterial pathogen associated with gastrointestinal infections, *C. difficile* is not limited to human populations and also has an impact on animals. The epidemiology of *C. difficile* in animals is an important area of study that focuses on understanding its presence, distribution, and dynamics within various animal species. Investigating the epidemiology of *C. difficile* in animals provides valuable insights into the transmission pathways and reservoirs of this bacterium. It helps to identify potential sources of infection and assess the risk of zoonotic transmission to humans.

The fact that *C. difficile* RT 078 and 078-like isolates (usually associated with ST 11) have been identified both in humans and animals has bolstered the hypothesis of a zoonotic origin of the infection. Although the transmission routes still remain unknown, there has been studies that provide evidence of the relationship of human and animal strains [15,16]. For instance, a high rate of *C. difficile* contamination with phylogenetically related strains was found both in the food chain and in farms, indicating a possible source of infection for humans [127]. In other study, the spread of clones included different continents, sometimes without any connection to healthcare facilities, suggesting that they were disseminated in the community through zoonotic or anthroponotic means over long distances. ST11 strain has a large pan-genome and contains various clinically significant antimicrobial resistance elements and prophages, which likely play a role in the successful global dissemination of this lineage that is of One Health significance [128].

However, the application of NGS techniques has allowed for the detection of subtle differences in strains that were previously considered indistinguishable using traditional typing methods. This kind of studies point in the direction of, rather than direct transmission, other shared infection sources being the origin of the infection. Like van Dorp et al. pointed out [19], even if direct transmission between hospital cases and animals are not related, community-acquired or foodborne transmission cannot be ruled out [129]. The existence of common sources of infection is another possibility that might explain these transmission events, making animals a reservoir for *C. difficile.*

*C. difficile* has been linked to livestock and companion animals on several occasions. *C. difficile* is considered an important cause of diarrhea in neonatal pigs. In particular, hypervirulent *C. difficile* RT 078 (ST 11) has been the most commonly isolated strain in pig farms, thus raising a public health concern [12]. *C. difficile* has also been documented in poultry, with notable occurrences of its presence in the fertilized grounds of specific chicken farms. This phenomenon led to the enduring colonization of the soil by *C. difficile* spores that exhibit a high degree of similarity to those commonly encountered in human patients. This scenario underscores the potential public health implications of the persistent presence of *C. difficile* in poultry and its potential role as a source of transmission to humans [130].

In horses, there have been inconsistencies in the reported prevalence and perceived impact of *C. difficile*, with prevalence ranging from 5% to 90%, although it seems to be higher in foals. At a molecular level, strains not associated with other species as well as those identified in other animals, the environment, and humans have been isolated [9].

Although less studied, *C. difficile* has also been studied in several wild [131] and exotic animals [132]. In general, these studies found a high prevalence of RT 078 and of uncommon RTs, varying between species [124,133]. The knowledge regarding the presence of *C. difficile* in wildlife and exotic pets is currently limited, mostly because of the diversity of these animal populations. The potential role of proximity to humans or other animals in the acquisition of *C. difficile* remains largely unexplored. Further studies should consider investigating whether certain animal species could serve as reservoirs of this bacterium or potential sources of infection. At present, the understanding of this aspect is lacking, and additional research is needed to shed light on the epidemiology of *C. difficile* in relation to wildlife and exotic pets.

Another important issue is the presence of *C. difficile* in the food chain. Although the possibility of *C. difficile* being a foodborne disease has already been studied without reaching a clear conclusion [129,134,135], new evidence suggests that *C. difficile* is present in the environment and along the food chain [136]. *C. difficile* contamination in slaughterhouses has been described [137,138] as well as in other products, like shellfish [139,140]. Furthermore, molecular relationship between strains isolated from animal feces and the strains found as contamination in the processing plant has been found [141].

While current evidence does not strongly indicate a high risk of infection, the possibility that slaughterhouses and the food chain may serve as a source of infection or act as reservoirs for *C. difficile* cannot be entirely disregarded. Although the exact contribution of the food chain to *C. difficile* transmission remains uncertain, the presence of characterized strains associated with animals and their occurrence in human CA-CDI cases warrants careful investigation. These analyses will help shed light on the potential role of slaughterhouses and other parts of the food chain in *C. difficile* epidemiology, ultimately leading to a better understanding of the dynamics of this pathogen and the implementation of appropriate preventive measures.

While our understanding of *C. difficile* epidemiology in animals is still evolving, recognizing the potential role of animals as reservoirs or sources of infection is crucial. Further research is needed to explore the transmission dynamics, host specificity, and impact of *C. difficile* in animal populations. By expanding our knowledge in this area, we can enhance the overall management and control of *C. difficile* infections, both in animal health and public health contexts.

## 4. Virulence Factors and Toxin Production

There has been previous research on the relationship between the levels of toxins produced by different strains of *C. difficile* and disease severity, but it has yielded contradictory results. However, a recent study that investigated the impact of different STs [142] found that, although there was no significant association between toxin levels and disease severity, ST 42 and ST 104, which produce high levels of toxins, had the most significant effects on cell proliferation, causing a reduction when compared to control cells. Additionally, ST 37 had a more severe impact on cell morphology. This highlights that not only strain characterization and toxin production but also host response play an important role in the onset of symptoms. Furthermore, it was found that stool samples containing *C. difficile* strains with ST 42 and ST 104 had higher levels of toxins.

The phylogenetic topology can be reflected in the PaLoc, which encodes toxins A (*tcdA*) and B (*tcdB*) and the negative regulator of toxin production, *tcdC*. Specifically, *tcdC* exhibited identical mutations across a group of analyzed ST 1 strains, as indicated by 1 and 18 bp deletions. In addition, fluoroquinolone resistance genes, particularly *gyrA*, exhibited uniformity in their sequences among analyzed ST 1 strains. On the other hand, these genes showed variations in their sequences when compared to those present in other STs. These findings suggest coevolution of the MLST genes with toxin genes and *gyrA*, indicating that the evolution of virulence and antimicrobial resistance in *C. difficile* may be linked to the evolution of the core genome [143].

In general, ST and virulence factors other than antibiotic resistance do not seem to be associated [144]. However, as for virulence genes, *agr* genes have been observed to have implications beyond toxin and virulence factors’ production regulation, although they have been found both in toxigenic and nontoxigenic strains. This type of gene has been implicated in the regulation of several characteristics related to *C. difficile* virulence, including flagellar biosynthesis, production of the toxin TcdA, and signaling proteins involved in cyclic dimeric GMP (c-di-GMP) signaling [145]. Notably, the distribution of *agr* gene variants differs among clades, with *agr2R*-positive strains primarily found in Clades 1 and 2, and STs carrying *agr2M* exclusively identified within Clade 4 [146]. It has also been hypothesized that host response, rather than ST or strain characteristics, plays a major role in the development of the disease [147].

In a recent study [85], the analysis of monolocus dendrograms revealed that the topologies derived from various housekeeping genes and certain virulence-associated genes, including *fbp68, groEL, tcdA, tcdB,* and *tcdB* loci, exhibited a consistent pattern, suggesting a possible co-evolutionary relationship. This implies that these genes have likely evolved together over time. However, the analysis also identified two discrepancies in the dendrograms. The *fliC* and *fliD* loci showed a global clustering pattern like that of the housekeeping genes, except for three isolates that were closely related to A-B+ isolates. On the other hand, the *cwp66* and *slpA* loci displayed a higher level of polymorphism compared to the other virulence-associated genes, resulting in a distinct dendrogram topology. Notably, certain isolates showed close relatedness in the *cwp66* and *slpA* trees but were distant in the other trees, indicating potential recombinational events or strong selective pressures affecting these gene clusters. As a conclusion of the study, the variability of the *slpA* gene, known to be associated with serogroups, seems to be influenced by both recombinational events and selective pressures. This suggests that the formation of serogroups in *C. difficile* is a result of the interplay between genetic recombination, environmental selection, and the polymorphic nature of the variable domain of the *slpA* gene.

In conclusion, the co-evolutionary relationship observed among certain housekeeping and virulence-associated genes suggests their concerted evolution over time. Discrepancies in dendrogram topologies indicate potential genetic recombination and selective pressures impacting gene clusters.

## 5. Antibiotic Resistance

Antibiotic resistance is a growing concern as it poses significant challenges to the effective treatment of CDI. Molecular typing techniques, such as whole-genome sequencing, PCR-based assays, and gene expression profiling, are essential tools for studying genetic resistance mechanisms and its link with phenotypical expression and the transmission of those genes. These techniques enable the identification of resistance genes, genetic variations, and mobile genetic elements, providing valuable insights into the molecular characteristics of antibiotic-resistant strains and aiding in the surveillance and control of resistant strains.

Regarding antibiotic resistance, the topic remains controversial as different studies have pointed in different directions. For example, some studies have found no relationship between ST and antimicrobial resistance, like no association between the presence of *erm*(B) and *tet*(M), MLST, and toxin phenotypes [148]. In other study, non-toxigenic *C. difficile* isolates had a lower presence of antibiotics resistance than its toxigenic pairs [149]. ST 2 isolates have been found more susceptible to ceftriaxone than other STs, suggesting a possible relation between ST type and ceftriaxone resistance [150]. However, in other studies, they found that the rates of clindamycin, tetracycline, and ampicillin resistance among ST 2 isolates were lower than those in other STs [151]. ST 81, which is closely related to ST 37, is capable of generating more spores and toxins and presents higher resistance rates to fluoroquinolones [57].

In China and other parts of Asia, ST 54 and ST 35 have been extensively associated with CDI as well as ST 37 [98,104,152]. ST 35 isolates have been found to be associated with tetracycline and erythromycin [149] and capable of causing outbreaks [153]. Additionally, genetic mutations related to glycometabolism, amino acid metabolism, and biosynthesis were associated with the transcription of *tcdR* and the expression of toxin repressor genes, *ccpA* and *codY*, suggesting their impact on protein activity stability, as this could cause instability and loss of function due to alterations in the protein structure [113]. Interestingly, in ST 37, despite exhibiting low toxin production (toxin A negative), there has been an alarming increase in antibiotic resistance within this particular strain, which raises concerns about its potential for widespread dissemination. The emergence of higher resistance rates in ST 37 strains underscores the need for vigilance and monitoring as these strains may pose an increasing threat and contribute to the spread of more resistant *C. difficile* strains [154]. High resistance to clindamycin has been described in this ST 37 [149]

Samples from Clades 1, 2, and 3 have been found to be more likely to exhibit reduced susceptibility to vancomycin, and moxifloxacin resistance was more frequently observed in samples collected from Europe and Asia compared to other continents, with no instances of moxifloxacin resistance identified in samples from Oceania [65].

The prevalence of multidrug resistance (MDR), defined as those isolates with non-susceptibility to three or more antimicrobial categories [155], was highest in Clade 4, with a rate over three times higher than in Clade 2. Three major epidemic *C. difficile* STs showed a strong association with specific antimicrobial resistance (AMR) determinants. ST 1 (Clade 2) was associated with fluoroquinolone resistance, ST 11 (Clade 5) with tetracycline resistance, and ST 37 (Clade 4) with macrolide–lincosamide–streptogramin B (MLSB) resistance and MDR. Among Clade 2 strains, a transposon labeled as *Tn6944* that carried *tetM* was identified [66]. In ST 42 (Clade 1), significantly higher rates of gene *erm (B)* (macrolide resistance) have been found compared with the others STs [156]. ST 3, also from Clade 1, was associated with a high rate of resistance to erythromycin and to moxifloxacin simultaneously. Association with decreased sensitivity to antibiotics is likely to favor its dissemination [112]. It is of special interest in the possibility of spread of pCD-METRO, a plasmid known to cause resistance to metronidazole, an antibiotic of special interest in the treatment of CDI. This plasmid has been found in strains isolated from the environment, as previously discussed [124].

In summary, the analysis of molecular characteristics of *C. difficile* strains reveals diverse patterns across different regions, genetic loci, and sequence types. The prevalence of specific STs varies geographically, highlighting regional differences in strain distribution, antimicrobial resistance profiles, and virulence factors. The association between STs and antibiotic resistance remains a topic of debate, with conflicting findings reported.

## 6. Conclusions and Future Directions

To sum up, the variety of methods used for the study of *C. difficile* allow researchers to adapt its projects to their resources and circumstances. However, there is a trend of shifting towards NGS methodologies as this technology becomes more affordable and provides high-resolution strain typing and detailed analysis, which can be easily integrated with epidemiological data to enhance outbreak investigations, track transmission routes, and identify potential virulence factors or antimicrobial resistance determinants.

Furthermore, integrating NGS data with phenotypic studies can provide a comprehensive understanding of *C. difficile*. By combining genetic information with phenotypic traits such as toxin production, sporulation ability, or antibiotic susceptibility, new information about correlations between genotypes and phenotypes would be obtained. This information could be used to improve treatments, shorten diagnosis times, and, in general, improve possible patient outcomes.

Besides, it would be also advisable to conduct studies in countries that do not have established *C. difficile* surveillance systems. By expanding research efforts to understudied regions, new insights into the global burden and distribution of *C. difficile* infections would be gained as well as into the identification of regional variations and antimicrobial resistance patterns, supporting the development of prevention and control measures.

In addition to NGS, other areas of research can contribute to our understanding of *C. difficile* epidemiology. Microbiota studies represent a promising field given the nature of *C. difficile*. The composition and dynamics of the gut microbiota play a crucial role in modulating susceptibility to *C. difficile* infection and disease outcomes. This kind of approach can give new insights into host–microbe interactions, identify potential biomarkers of susceptibility or resistance, and explore novel approaches for preventing or treating CDI. It can also be linked to new ways of treatment, like fecal microbiota transplantation.

In addition, adopting a One Health approach is another promising take for future studies. *C. difficile* is not limited to human populations and can be found in animals, food, and the environment. There is evidence that the epidemiology of *C. difficile* has changed, therefore making it important to investigate new routes of transmission, which include a possible zoonotic element or the existence of higher rates of CA-CDI than traditionally reported. Investigating the epidemiology of *C. difficile* across different species and environments can help elucidate transmission dynamics, reservoirs, and potential sources of infection.

Lastly, studying host–pathogen interactions in CDI could prove to be an important area of investigation. Understanding the mechanisms underlying host susceptibility, immune response, and disease severity can help with the development of targeted therapeutics and better treatments. As exposed previously, strain characterization alone does not explain the development of CDI, suggesting that host response may play a significant role in the disease process.

By focusing on these future perspectives, researchers can contribute to a deeper understanding of *C. difficile* epidemiology, enhance our ability to prevent and control CDI, and improve patient outcomes.

## Figures and Tables

**Table 1:** Comparison of molecular techniques used for *C. difficile* characterization. Most common advantages and disadvantages are listed.

Technique	Description	Advantages	Disadvantages
Banding pattern-based analysis			
PCR Ribotyping	It analyzes the diversity of the 16S-23S intergenic spacer region through gel electrophoresis	- Widely used and well-established technique - Standardized protocols available - Rapid and cost-effective - Good inter-laboratory comparability	- Moderate discriminatory power compared to other methods - Interpretation of complex banding patterns can be challenging and subjective - Limited resolution at strain level for some closely related isolates - It cannot be obtained through WGS
PFGE (Pulsed-field gel electrophoresis)	It analyzes the banding patterns of fragmented DNA with an alternating electric field to separate larger fragments	- High discriminatory power - Widely used and well-established technique - Standardized protocols available - Compatibility with existing databases	- Time-consuming and labor-intensive - Skilled personnel and specialized equipment are required - Interpretation of complex banding patterns can be subjective - It cannot be obtained through WGS
REA (Restriction endonuclease activity)	It analyzes patterns of restriction enzyme digested DNA	- High discriminatory power - Suitable for outbreak investigations	- It requires optimization for each target gene or region - High manual labor and subjectivity in pattern interpretation - Lack of standardization - It cannot be obtained through WGS
MLVA (Multilocus variable-number tandem-repeat analysis)	It analyzes variations in tandem repeats within multiple loci	- High discriminatory power - Suitable for outbreak investigations - Rapid and cost-effective - Easy interpretation of results	- Relatively low resolution compared to other methods - Dependent on the choice of loci and primers
Toxinotyping	Analyzes the polymorphisms in fragment length of PaLoc (REFL-PCR)	- It provides information on the toxin variants of *C. difficile* - Well-stablished technique	- Limited discriminatory power compared to other methods - Interpretation can be challenging due to the presence of multiple toxin genes and variants - Irregular distribution of PaLoc across phylogenetic groups - It cannot be obtained through NGS
Sequence-based analysis			
MLST (Multilocus sequence typing)	It analyzes sequences of selected housekeeping genes	- Highly portable results across laboratories - It allows comparison of data between studies - Phylogenetic studies are possible	- It requires DNA sequencing, which can be costly - Limited discriminatory power compared to other methods - Targeted loci may not reflect the entire genome diversity
WGS (Whole-genome sequencing)	It analyzes sequencing of large amounts of DNA	- High-resolution genomic analysis - It can trace phylogenetic relationships - It enables identification of genetic variants and resistance markers - Potential for discovering new virulence factors	- Costly - Specialized bioinformatics analysis is required - Longer turnaround time for analysis - Data storage and management can be challenging

**Table 2:** Ribotype and sequence type relationship of commented *C. difficile* strains, including the reference sequence type (in bold) for each clade. Cryptic clades do not have a reference type.

Clade	Sequence Type	Ribotype	References
1	2	005, 020/014, 015, 069, 076, 095, 220	[20,48,49]
	**3**	001, 009, 055, 072, 077, 115, 262, 305	[48,49]
	10	015	[48]
	17	018, 052	[48,49]
	33	014/020, 064, 216, 369	[48,49,50]
	34	056	[48]
	35	002, 046, 220	[48,51]
	42	106, 118, 174	[48,49]
	44	015, 062	[48,49]
	45	013, 017	[48,50]
	54	012, 014/020	[48,52]
2	**1**	002, 003, 016, 027, 036, 176	[48,49,52]
	41	46, 106, 156, 164, 194, 208, 209, 244, 321	[48,49,50,53]
3	**5**	023, 063, 069, 122, 438	[49,54,55]
	22	023	[54]
4	**37**	017, 047	[49,56]
	81	PKI-017, A	[57,58,59]
5	**11**	033, 045, 066, 078, 126, 127, 193, 193, 237, 280, 281	[48,49,53,60,61]
I	200	Not studied	[62]
I and II	946, 947 and 948	151	[63]
II	181	Not studied, phylogenetically similar to NML211	[62,64]
III	369	Not studied	[62]

## Data Availability

No new data were created or analyzed in this study. Data sharing is not applicable to this article.
